# Dark-Field Microscopic Study of Cellular Uptake of Carbon Nanodots: Nuclear Penetrability

**DOI:** 10.3390/molecules27082437

**Published:** 2022-04-09

**Authors:** Wendi Zhang, Zuowei Ji, Zheng Zeng, Anitha Jayapalan, Bhawna Bagra, Alex Sheardy, Peng He, Dennis R. LaJeunesse, Jianjun Wei

**Affiliations:** 1 Department of Nanoscience, Joint School of Nanoscience and Nanoengineering, University of North Carolina at Greensboro, Greensboro, NC 27401, USA; wendyzhang2013@sina.com (W.Z.); jizuowei@cuhk.edu.cn (Z.J.); hdzengzheng@163.com (Z.Z.); a_jayapa@uncg.edu (A.J.); bhawnacct@gmail.com (B.B.); alex.sheardy@gmail.com (A.S.); drlajeun@uncg.edu (D.R.L.); 2 Department of Chemistry, North Carolina Agricultural and Technical State University, Greensboro, NC 27411, USA; phe@ncat.edu

**Keywords:** nitrogen-doped carbon nanodots, cellular uptake, light scattering, enhanced dark-field microscopy, hyperspectral imaging, nuclear penetration

## Abstract

Carbon nanodots are fascinating candidates for the field of biomedicine, in applications such as bioimaging and drug delivery. However, the nuclear penetrability and process are rarely studied and lack understanding, which limits their applications for drug carriers, single-molecule detection and live cell imaging. In this study, we attempt to examine the uptake of CNDs in cells with a focus on the potential nuclear penetrability using enhanced dark-field microscopy (EDFM) associated with hyperspectral imaging (HSI) to quantitatively determine the light scattering signals of CNDs in the cells. The effects of both CND incubation time and concentration are investigated, and plausible nuclear penetration involving the nuclear pore complex (NPC) is discussed. The experimental results and an analytical model demonstrate that the CNDs’ uptake proceeds by a concentration-dependent three-stage behavior and saturates at a CND incubation concentration larger than 750 µg/mL, with a half-saturated concentration of 479 μg/mL. These findings would potentially help the development of CNDs’ utilization in drug carriers, live cell imaging and other biomedical applications.

## 1. Introduction

Carbon nanodots (CNDs) are composed of functional groups, amorphous carbon frames and polyatomic carbon domains [1,2]. CNDs can be synthesized by the techniques of microwave irradiation, hydrothermal or solvothermal treatment, laser ablation, electrochemical carbonization and chemical ablation [3,4], as well as ultrasonic irradiation [5,6]. CNDs are reported to have potential applications in photoelectric devices [7], chemical sensing, [8] bioimaging [9], antibacterials, [10] and biomedicine owing to their superior water solubility, biocompatibility, tunable photoluminescence and opto-electronic properties [11,12,13].

Many efforts have been made to investigate CNDs for biomedicine applications such as drug delivery, radical scavenging, regulating oxidative stress in cells, and live cell sensing/imaging due to their small size, hydrophilicity and readiness for functionalization [11,14,15,16,17,18,19,20,21,22,23]. Wu et al. developed multi-functionalized CNDs that target lung cancer cells and showed good anti-cancer effects [24]. Khan et al. reported single-molecule detection in the nucleus using CNDs [25]. Dekaliuk et al. used CNDs to visualize cellular functioning processes such as apoptosis [26]. For such biomedical applications, the interaction of the CNDs with cells is critical for improving the performance. Recent reports concluded that targeted nuclear delivery in cells could show enhanced therapeutic effects [27]. Some kinds of nanoparticles sized between 10 and 100 nm (gold, silver, silicon, etc.) have been studied and nanoparticles with a diameter of 50 nm showed very high cell uptake [28,29,30,31]. Due to a good fit with the cellular pore size, smaller nanoparticles with a size of 5 nm to 10 nm (gold, silver, silicon, etc.) obtain a higher cellular penetration [32,33,34]. More importantly, small nanoparticles can be located in endosomes, lysosomes, cell cytoplasm and the nucleus [35]. While there has been tremendous progress in research on CNDs for biomedicine applications, there is a lack of a comprehensive understanding of how CNDs enter and leave the cells.

In terms of techniques to study the cell–nanoparticle interaction, fluorescence microscopy is a useful tool to track nanoparticle uptake and trafficking route [36]. This technique uses a specific excitation wavelength for a sample which is fluorescently marked or intrinsically fluorescent to generate the emission signal [37]. Since CNDs can provide a fluorescence emission ranging from blue to red by adjusting the preparation conditions, increasing the surface oxidation degree, doping different elements, or controlling the size, cellular nucleus targeting or mitochondria targeting using CNDs has been studied by fluorescence microscopy [38,39,40,41,42,43,44,45]. The functionalization of CNDs with specific biorecognition ligands provides precise in vitro and in vivo bioimaging applications [46]. Recently, Hua et al. successfully imaged nuclei in cells when stained with green fluorescent CNDs or CND–protoporphyrin IX conjugates using a confocal technique [47]. However, the fluorescent labels might be quenched or bleached during the measurement [48], resulting in the loss of the photoemission signal [49]. In addition, cross-talk may happen between the different channels when multiple channels of a microscope are used to observe the nanoparticles and cellular compartments [50]. In contrast, the cross section of light scattering from metal nanoparticles (gold, silver, etc.) is orders larger than the cross section of photoemissions from typical fluorophores or quantum dots [51,52]. Advanced light-scattering microscopy techniques may also provide an alternative means for the cellular uptake of CNDs and nuclear penetration. It would be of great significance to better understand the nuclear penetrability of CNDs in terms of the development of the biomedical applications of CNDs as drug carriers, living cell imaging and detection, and in vivo studies.

In this work, we use enhanced dark-field microscopy (EDFM) assembled with a hyperspectral imaging (HSI) system for tracking the CNDs in cells (Human A549 cells) and to achieve a better understanding of CNDs’ cellular uptake, specifically the nuclear penetrability. The methodology of EDFM coupled with HSI is capable of visualizing nanoscale objects with similar refractive indexes as the background, because this method can provide high contrast based on the indirect illumination of the specimen and the resultant reflected or elastically scattered light of the objects upon interaction with the sample [53]. While the cellular uptake of other nanoparticles, such as gold and crystalline quantum dots, has been well investigated with regard to the effects of size, surface properties and morphology, studies addressing the nuclear penetrability of CNDs in cells are very few. In this study, we examine the effects of the incubation time and concentration of nitrogen-doped CNDs on the penetration into the nucleus of a cell using hyperspectral microscopy technology to quantitatively describe the light signals in the nucleus with respect to temporal and spatial aspects. The nuclear penetrability is corroborated by confocal imaging. The mechanism of CND penetration is discussed based on the concentration-dependent manner. This research, to the best of our knowledge, is the first to use EDFM coupled with HSI for investigating the cellular uptake and nuclei penetration of CNDs.

## 2. Results

### 2.1. Synthesis and Characterization of CNDs

**Figure 1:**
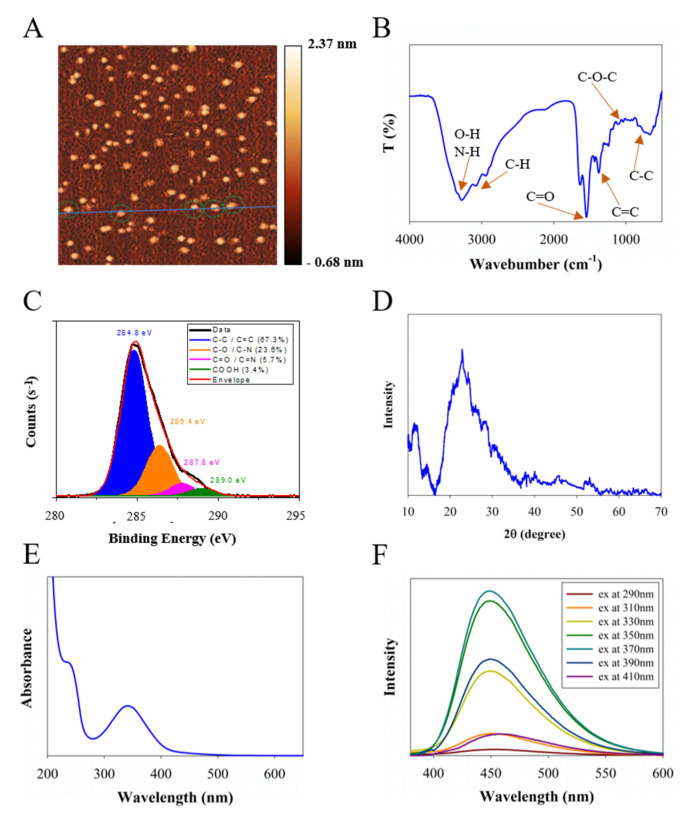
CNDs characterization: (**A**) AFM topography image, (**B**) FTIR spectra, (**C**) XPS signal (C 1s), (**D**) XRD data, (**E**) UV−Vis absorption spectra, and (**F**) fluorescence emission spectra.

The CNDs in this work were synthesized using a microwave-assisted method with precursor molecules of ethylenediamine and citric acid, which were dissolved in deionized water. The purified product was characterized by employing a variety of microscopic and spectroscopic tools. Atomic force microscopy data (Figure 1A) with associated height profile analyses (Figure S1) indicate that the CNDs have an average size of about 2 nm, which is verified by the transmission electron microscope data with associated diameter profile analyses (Figure S2). According to the FTIR spectra (Figure 1B), ν(O-H) and ν(N-H) are presented corresponding to the presence of broad bands (from 3100 cm^−1^ to 3400 cm^−1^), which help to support the hydrophilicity and stability of CNDs in aqueous solution [54]. The surface charge of CNDs is slightly negative by the zeta potential measurement (−8.1 mV). The FTIR signals at 690 (C-C), 1185 (C-O), 1375 (C=C), and 1550 (C=O) cm^−1^ can also be assigned, respectively [20,55]. The XPS data (Figure 1C) indicate components of C-C and C=C (284.8 eV, 67.3%), C-O and C-N (286.4 eV, 23.6%,), C=O and C=N (287.8 eV, 5.7%), and COOH (289.0 eV, 3.4%) [13]. The atomic ratio of C:N:O is estimated to be 78:16:6 by the survey XPS spectrum analysis associated with the XPS spectra of N 1s and O 1s (Figure S3). XRD confirms the graphite structure due to the main diffraction peak at 22.8° with a full width at half maximum (FWHM) of about 4.1° (Figure 1D). UV-Vis (Figure 1E) shows two main absorption features, the π-π* transitions of C=C and n-π* transition of C=O, which can be seen at about 235 nm and 350 nm, respectively [56]. Figure 1F shows the fluorescence emission spectra under different excitation conditions (λ_ex_ in the range of 290–410 nm). At an excitation wavelength of 370 nm, the peak emission presents at 450 nm. The highest quantum yield obtained is 61 ± 3% and the fluorescence mechanism was well studied previously [8,13,57]. Note that the concentration of CNDs used for UV-Vis and fluorescence is 50 μg/mL. A photobleaching experiment was conducted and results are shown in Figure S4. The photoluminescence of carbon nanodots exhibits a slight reduction under Xe lamp irradiation with high light power from 112 W to 168 W for 30 min, which indicates their good photostability.

### 2.2. Dark-Field Microscopy and Hyperspectral Imaging of Cells with CND Incubation at Different Time

The A549 cells were first studied using dark-field microscopy and hyperspectral imaging with a concentration of 750 μg/mL of CNDs at different incubation times as a comparison to the cells without CND incubation. The CytoViva imaging system was adjusted to provide high-signal-to-noise optimized dark-field images by oblique angle lighting, and the reflecting/scattering light intensity can be obtained from the images [56]. Note that all the images were obtained using the same light exposure time with the 100× objective to cover the measured area (X × Y) of 20 μm × 20 μm of a total pixel of 400 × 400, and set at the same Z profile for the imaging across the nucleus area. Different from the super-resolution fluorescence microscopy, transmission electron microscopy, atomic force microscopy, or scanning electron microscopy used to probe the cellular interactions with nanoparticles, light reflection and scattering spectra from the cells can be automatically captured by the integrated CytoViva system to record and determine whether CNDs are present in the nucleus region in the cells. Each pixel of a hyperspectral image provides the complete reflectance and scattering spectral response of that pixel’s spatial area within the visible to near infrared (NIR) range, of 400–1000 nm wavelength.

**Figure 2:**
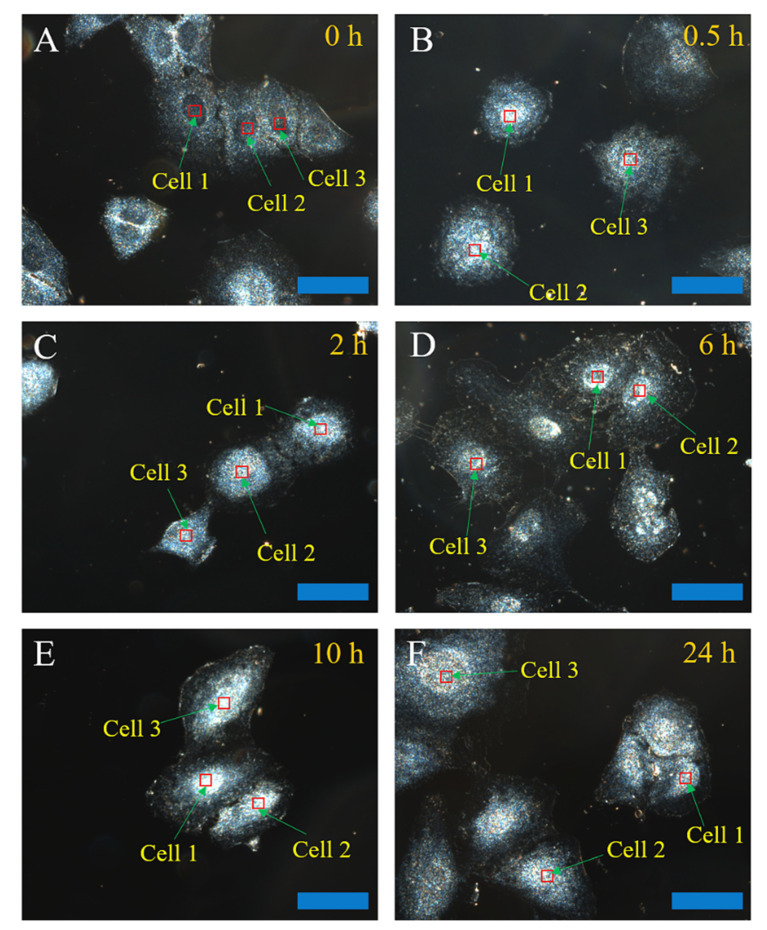
Dark-field images of cells incubated without CNDs (**A**) or with 750 μg/mL of CNDs for different incubation times ranging from 0.5 h to 24 h (**B**–**F**). All of the scale bars are 4 μm.

To investigate the CND uptake and nuclear penetration, the A549 cells were incubated by CNDs with a concentration of 750 μg/mL for various lengths of time, ranging from 0.5 h to 24 h. Thereafter, the cells were washed with PBS (pH 7.4) twice for each well and then fixed with 4% paraformaldehyde solution for 15 min at room temperature. Figure 2A shows the dark-field image of the cells without CND incubation. The nucleus structure can be clearly seen in the image. Figure 2B–F show the dark-field light scattering images after CND incubation at different times. It is expected that, if the CNDs can enter the nucleus, the CNDs will lead to a bright part in the nucleus, as shown in the dark-field images due to light scattering/reflection, similar to a previous report using peptide-modified gold nanoparticles in CytoViva imaging results [58]. In this study, the white spots of the nucleus in the dark-field image represent the CNDs’ localization with the peak of reflection spectra at around 580 nm from the integrated CytoViva hyperspectral imaging. As mentioned in the characterization section, the precursors of ethylenediamine and citric acid introduce carboxyl groups and negative charges to the surfaces of CNDs, making the distribution of CNDs more uniform in the incubation process. The presence of CNDs in the nucleus’ structure were characterized by the light intensity of the nucleus (indicated in Figure 2). In Figure 2B,C, slightly brighter light signals form at the center of the cells and the light signal is uniformly distributed across the whole cell, suggesting the starting of CND nuclear penetration with an incubation time of 0.5–2 h. As the CNDs’ incubation time increases from 6 h to 24 h (Figure 2D–F), the light signal in nuclear regions becomes stronger.

**Figure 3:**
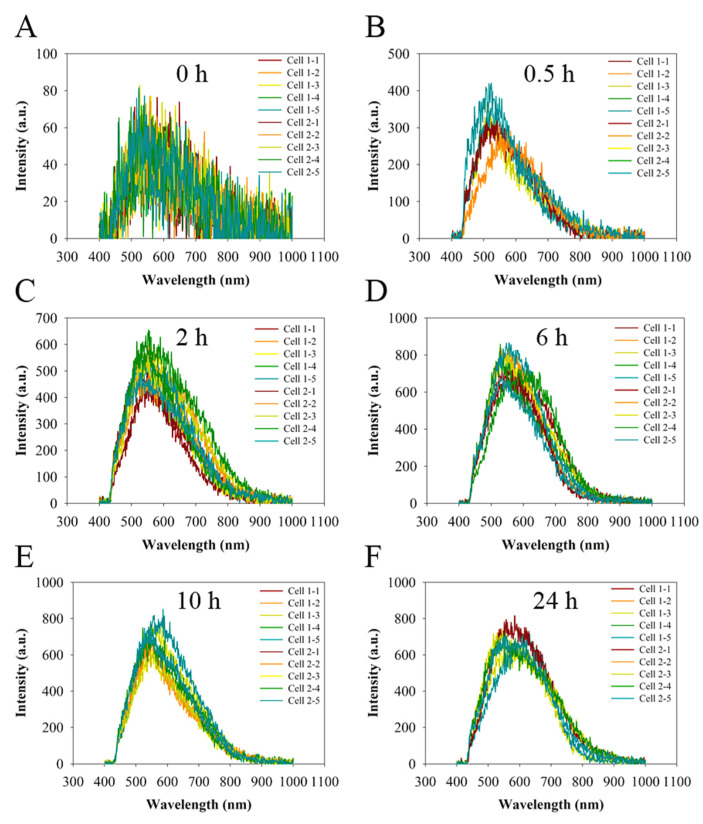
Light intensity spectra (a total of 34 measurements) of the nuclear area of the cells without CNDs (**A**) or with 750 µg/mL of CNDs for different incubation times from 0.5 h to 24 h (**B**–**F**).

**Figure 4:**
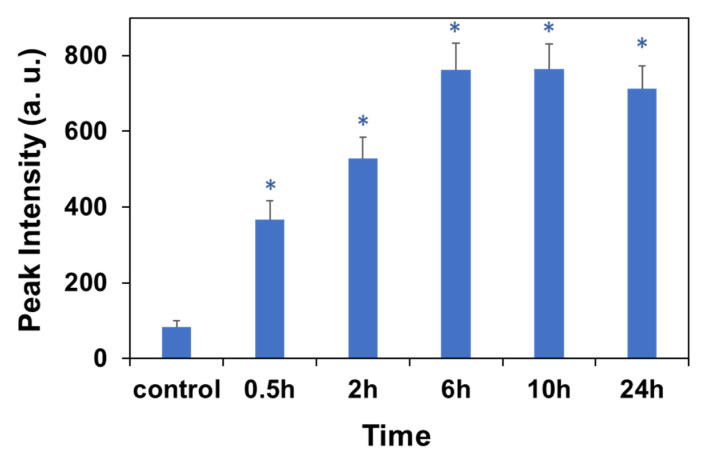
Light intensity of nucleus region of cells with 750 µg/mL of CNDs incubation as a function of time for incubation. The error bar shows the standard deviation. * Stands for a significant difference from the control (0 mg/mL) (*p* < 0.05) via a statistical analysis.

In order to quantitatively determine the light signals in the nucleus, we extracted the hyperspectral signal via the ENVI software (Figure 3) based on 34 points at the nuclear area of cells (represented in Figure 2). It is not surprising that the background spectra (Figure 3A) of the cells have a similar reflection/scattering peak at around 550 nm to the spectra of cells treated with CNDs because of the similar refractive index to the cell tissue. The average peak light intensity is plotted as a function of the incubation time, as shown in Figure 4. The light intensity shows a monotonic increase with the increase in the incubation time up to 6 h, then shows a slight decrease to reach a plateau (Figure 4), a feature that is consistent with another report by a fluorescence imaging study [59]. After incubation for 0.5 h, the nuclear light intensity is enhanced by the uptake of CNDs to be about 380 a.u. in comparison to the background light intensity of about 89 a.u. obtained from the cells without CND incubation at the same condition. The light intensity increased up to 770 a.u. with a CND incubation time of 6 h. In a control experiment, the hyperspectral signal via the ENVI software was conducted focusing on the nucleus region without CND incubation using the same time periods. Since the control cells without CND incubation show a negligible light scattering increase for the time periods (Figure S5), one can conclude that the light intensity increase in the nuclear region of the cells with CND incubation implies the nuclear penetration of CNDs.

### 2.3. Light Intensity Measurements under CNDs Incubation with Different Concentrations

**Figure 5:**
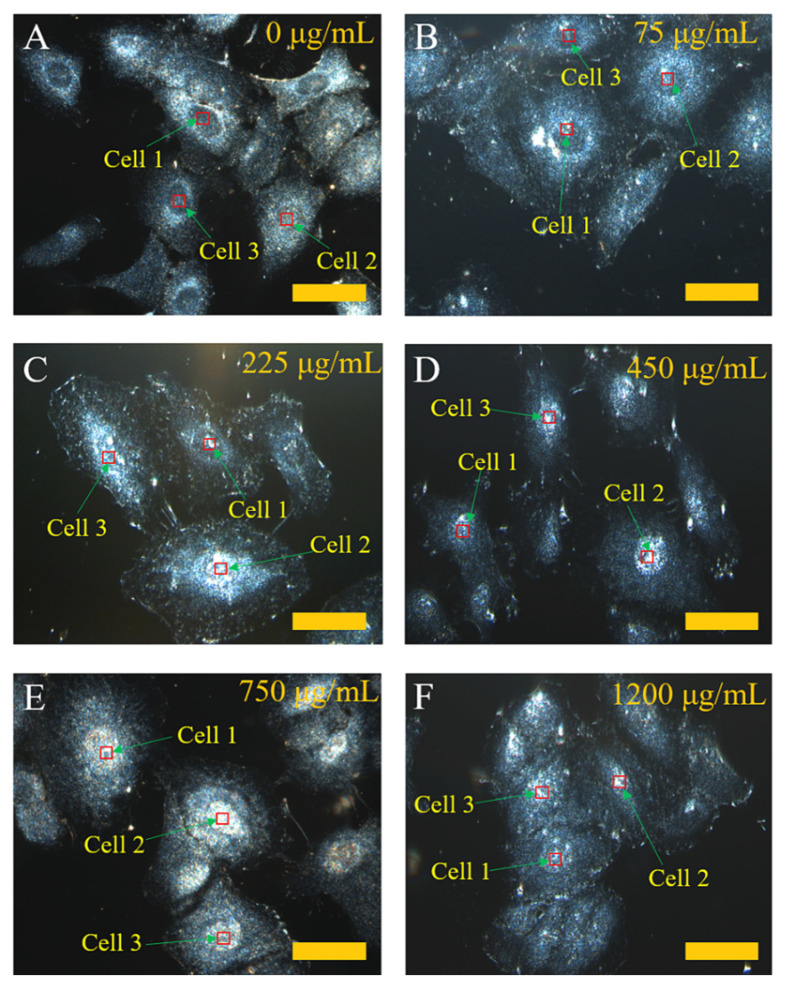
Dark-field images of cells incubated without CNDs (**A**) or with CNDs for 24 h using different concentrations ranging from 75 μg/mL to 1200 μg/mL (**B**–**F**). All the scale bars are 4 μm.

To investigate the effect of the CNDs’ concentration on their penetration into the nucleus, the cells were incubated for 24 h with different CND concentrations. Figure 5 shows the dark-field images of a cell with the incubation of CNDs at concentrations ranging from 0 μg/mL to 1200 μg/mL. At a CND concentration of 75 μg/mL, the average light signal at the boundary region of the nucleus is higher than that of the nucleus region (Figure 5B). It is worth noting that the very bright spots in Figure 5B Cell 1 may be due to some aggregates of CNDs outside the nucleus. When the CND concentration increases, the light signals increase at the center of most cells and are eventually distributed in the cells (Figure 5C–F). For the incubation at 750 and 1200 μg/mL of CNDs for 24 h, the dark-field images show a similar brightness of light signals with an insignificant difference. In this study, the viability of cells is important to assure that the cells for imaging are alive. Hence, we conducted cytotoxicity measurement by the MTT assay for A549 cells incubated with different concentrations of CNDs for 24 h (Figure S6). For the highest concentration at 1200 µg/mL CND incubation, the viability of the cells remains ~75%. These observations indicate that the CNDs exhibit good biocompatibility.

**Figure 6:**
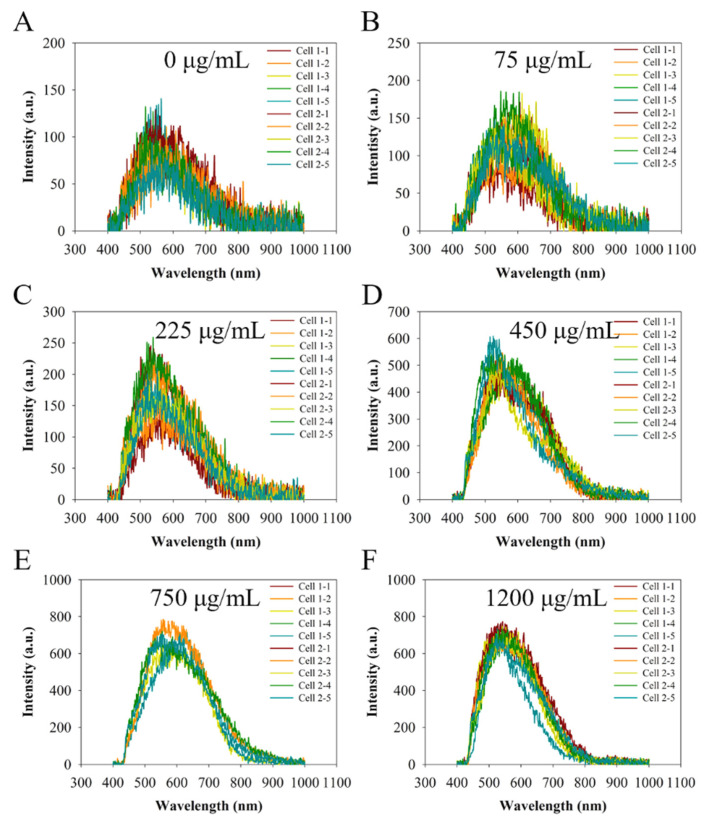
Light intensity measurements of cells (34 measurements) incubated without CNDs (**A**) or with CNDs for 24 h using different concentrations ranging from 75 μg/mL to 1200 μg/mL (**B**–**F**).

**Figure 7:**
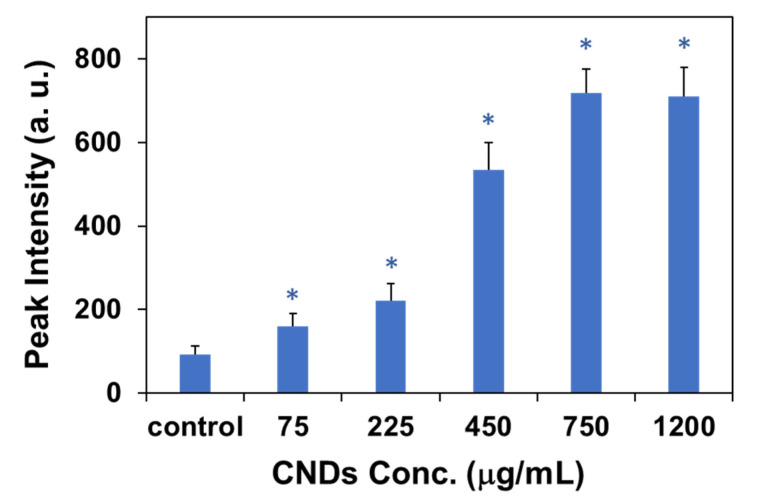
Light intensity of nucleus region of cells with 24 h CND incubation as a function of concentration for incubation. The error bars represent standard deviation. * Stands for a significant difference from the control (0 mg/mL) (*p* < 0.05).

The quantitative determination of the light signals (from the 34 points) in the nucleus (Figure 5) was further performed by analyzing the hyperspectral spectra via the ENVI software. The results are presented in Figure 6. The light intensity shows a monotonic increase with the increase in the incubation concentration of CNDs up to 750 μg/mL (Figure 7). At the range of CND concentration from 0 to 225 μg/mL, the peak light intensity is gradually increased from 100 a.u. to 220 a.u. At an incubation concentration of 450 μg/mL or higher, the light intensity increases dramatically to >500 a.u. (max), suggesting a great amount of CND penetration into the nucleus of the cells. However, a feature of constant light intensity is observed when the CND concentration for incubation is beyond 750 μg/mL, suggesting a saturation of CND penetration with the incubation concentration of 750 μg/mL or higher.

To confirm the localization of CNDs in the nuclei of the cells, a fluorescence confocal technique was used to image the cells stained with Mitotracker Red and the fluorescence of the CND uptake in A549 cells at a concentration of 1000 μg/mL, respectively. As the CNDs are blue fluorescent, DAPI dye was not chosen to stain the nuclear area. Instead, the Mitotracker Red labeling the mitochondria is used to discriminate the nuclear region inside cells. Figure S7 shows confocal images of Mitotracker Red labeled cells, the CND fluorescence in cells, and merged Mitotracker Red and CND fluorescence in cells. It clearly shows the subcellular localization of the CNDs in the nuclear region, though some CNDs are found in the cytoplasm, corroborating the nuclear penetration.

## 3. Discussion

**Figure 8:**
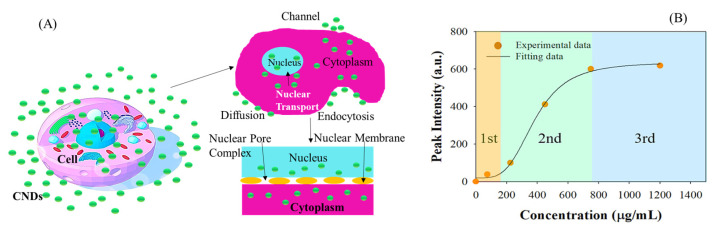
(**A**) Schematic view of the nuclear penetration process of CNDs (the drawing is not to scale). (**B**) The concentration-dependent data analysis of the nuclear penetrability of CNDs and a three-stage model fit.

Three basic mechanisms have been proposed for interpreting how nanoparticles enter cells: endocytosis, penetration through channels, and direct diffusion across the plasma membrane [60,61]. According to the characteristics of the CNDs’ structures, it is plausible that the penetration of CNDs into the cells involves the synergistic effects of direct diffusion across the plasma membrane, receptor-mediated and/or fluid-phase endocytosis, and passing through the channels in the plasma membrane, followed by CND nuclear penetration through the nuclear pore complex (NPC), one kind of large proteinaceous assembly. Figure 8A schematically shows a proposed penetration process. The nuclear membrane contains the NPCs, which can function as selective channels for the transport of molecules and the mediation of nucleocytoplasmic exchange [62,63].

Conforming to the experimental results, the nuclear penetration of CNDs presents dependence on the incubation concentration (Figure 7). Note that the intensity data were obtained by subtracting the background signal without CND incubation, the net intensity representing the concentration of CNDs in the nucleus. Herein, for a better understanding of the CND nuclear penetrability, we build a model to delineate the relationship between the light intensity and incubation CND concentration. In this analysis, it should be noted that all equations are dimensionless, since we use the relative values of the parameters that are normalized to their highest value. In this case, the rate of CND penetration can be expressed:

*dI*/*dt* = *α*(*C* − *C_s_*), (1)

where *I* is the light intensity and *dI/dt* is defined as the rate of nuclear penetration of CNDs, *α* is the assumed penetration flux, *C* is the incubation concentration, and *C_s_* is the saturation CND concentration at which CNDs in and out of the nucleus are balanced [32]. A nonlinear exponential relationship could be built for the nuclear penetration of CNDs regarding the radius of the nucleus structure for a single cell [50,64,65]:

*C* = *β*exp(*εr*/*I*), (2)

where *β* and *ε* are the characteristic constants to fit the nonlinear exponential relationship with the light intensity and incubation concentration, respectively, and r is the radius of the nucleus structure for a single cell.

Based on the nonlinear relationship between incubation concentration and light intensity, the saturation of light intensity at dynamic balance (*I_max_*) is thus given by the equation:

*I_max_* = *εr*/(ln(*C_s_*/*β*)), (3)

Hence, based on the measurements results, we can obtain the saturation of light intensity and the characteristic constants to determine the nonlinear relationship between incubation concentration and light intensity.

Furthermore, to analyze the nuclear penetration process of CNDs, the following equation was used to conduct the best fit of the light intensity versus incubation concentration data:

*I* = *I_min_* + (*I_max_* − *I_min_*)/(1 + 10^(*C*_0.5_−*C*)^)(*C* > 0), (4)

Figure 8B shows an *I_min_* of about 22 a.u., *I_max_* of about 639 a.u. and a half saturation incubation concentration (*C*_0.5_) of ~479 μg/mL.

The model suggests the three-stage concentration-dependent nuclear penetrability of the CNDs. The first stage of the nuclear penetration happens at CND concentrations below 160 µg/mL with a slow penetration process; the second stage of fast CND penetration ranges from 160 to 750 μg/mL concentration of CNDs; and the third stage occurs at a CND concentration of >750 μg/mL. For the concentration between 75 and 750 μg/mL CNDs, the light intensity increases and presents in a concentration-dependent manner until the CND penetration reaches a plateau in terms of light-scattering signal at a concentration higher than 750 μg/mL of CND incubation. Corroborating the above-proposed nuclear penetration process, the first stage at low concentration involves CND penetration on the nuclear-side boundary layer of the NPC. The second stage engages an association with importins to transport the collective CNDs at increased concentrations with larger CND aggregates. Following transport, the CNDs are comprehensively distributed in the nucleus due to dissociation between the aggregated CNDs and importins originating from the conformation change in the importins [66]. The third-stage process at higher CND concentrations is the “cessation” of CND penetration into the nucleus even with increasing CND concentrations due to the “saturation” of CNDs in the nucleus.

## 4. Materials and Methods

### 4.1. Synthesis of CNDs

CND synthesis was similar to that of a previous study [8,67]: precursors (ethylenediamine + citric acid) were used to synthesize nitrogen-doped CNDs by a microwave-assisted method. Briefly, citric acid (0.96 g, 99%, ACROS Organics, Morris Plains, NJ, USA) and ethylenediamine (1.0 mL, 99%, Alfa Aesar, Haverhill, MA, USA) were mixed together in 1.0 mL deionized water to form a homogenous solution. Then, a microwave synthesizer (CEM Corp 908005, Matthews, NC, USA) was used to heat the solution at 300 W for 15 min. Dialysis (1000 MWCO, Fisher Scientific, Pittsburgh, PA, USA) against deionized water (three times) was used to purify the reddish-brown solution for 24 h. Lastly, the resultant solution was freeze-dried (FreeZone 6, Labconco, Kansas City, MO, USA) for 24 h to obtain the solid sample.

### 4.2. Characterization of CNDs

Atomic force microscopy (AFM, 5600LS Agilent, Cary, NC, USA) and a transmission electron microscope (FEI Tecnai G2 F20, Hillsboro, Oregon, USA) were used to test the size and morphology of the CNDs, and a zeta potentiometer (Malvern Zetasizer ZEN3600, Malvern, United Kingdom) was used for the determination of the charge of the CNDs. To study the chemical structure and elemental content of the CNDs, Fourier transform infrared spectroscopy (670 FTIR, Varian, Crawley, United Kingdom), X-ray photoelectron spectroscopy (XPS, Thermo Fisher ESCALAB 250 Xi, Waltham, MA, USA) and X-ray powder diffraction (XRD, Agilent Technologies, Cary, NC, USA, Oxford Gemini) were performed. The optical properties of the CNDs were studied by UV-Vis spectroscopy (Cary 6000i, Agilent, Cary, NC, USA) and fluorescence spectroscopy (Cary Eclipse, Agilent, Cary, NC, USA).

### 4.3. Cell Culture

Human A549 cells (ATCC, CCL-185) were seeded in the growth medium F-12K (Kaighn’s Modification of Ham’s F-12 medium, Thermo fisher, Waltham, MA, USA) (ATCC, 30-2004) supplemented with 10% fetal bovine serum (Sigma Aldrich, Burlington, MA, USA) and 1% streptomycin–penicillin (Fisher Scientific, Pittsburgh, PA, USA) in a T-75 flask at 37 °C and 5% CO_2_ in a cell incubator for 70~90% confluence. Then, the cells were grown on coverslips in a 12-well plate in a cell incubator at 37 °C and 5% CO_2_ for 24 h to make cells fully spread. After that, the cells were treated with 750 μg/mL of CNDs for different lengths of time (0 h, 0.5 h, 2 h, 6 h, 10 h and 24 h). For the concentration-dependent experiments, the cells were treated for 24 h at different concentrations (0 μg/mL, 75 μg/mL, 225 μg/mL, 450 μg/mL, 750 μg/mL and 1200 μg/mL).

### 4.4. Sample Fixation

After treatment of CNDs for different times and at different concentrations, the cells were washed by PBS (Thermo Fisher, Waltham, MA, USA, pH 7.4) twice for each well and then the cells cultured on the coverslips, which were fixed with 4% paraformaldehyde solution for 15 min at room temperature. The paraformaldehyde was removed by PBS washing (three times). Coverslips were mounted on glass slides by using a small drop of mounting media (Polysciences, Inc., Warrington, PA, USA). The samples were stored at 4 °C in a dark environment before imaging.

### 4.5. Dark-Field Microscopy with Hyperspectral Imaging

The enhanced dark-field microscope (CytoViva, Inc., Auburn, AL, USA) can enable users to image nanomaterials by improving signal-to-noise by over ten times compared with a standard dark-field microscope. This microscope was used to observe CND uptake by A549 cells. The images were obtained with a 60× immersion oil objective and then the hyperspectral signal was acquired in the same position of the glass slide by a CCD camera under the white light source. For the data analysis, the hyperspectral signal was output via the ENVI software and optical images were obtained by the Ocular software.

### 4.6. 3-(4,5-Dimethylthiazol-2yl)-2,5-diphenyltetrazolium Bromide (MTT)-Based Assay

Cells were firstly seeded in a tissue-culture-treated 24-well plate and incubated for 24 hr. Then, the cells were further incubated with different concentrations of CND suspensions ranging from 75 to 1200 µg/mL for another 24 hr. After rinsing the cells with phosphate-buffered solution (PBS), 0.2 mg/mL MTT (99%, Fisher Scientific, Pittsburgh, PA, USA) solution was added to the cells and incubated for an additional 2 hr. Afterwards, dimethyl sulfoxide (DMSO) was used to dissolve the formazan crystal after rinsing the cells with PBS. Then, a BioTek microplate reader was used to measure the absorbance of each well at a wavelength of 570 nm.

### 4.7. Confocal Imaging

Briefly, the A549 cells were firstly plated on glass coverslips in a 12-well tissue culture plate and incubated for one day. Then, CND suspensions of concentration 1000 µg/mL were added to replace the old medium. After 24 h exposure, the cells were rinsed with PBS at least three times. Then, the cells were stained with MitoTracker Red CMXRos (Fisher Scientific, Pittsburgh, PA, USA) for 10 min (0.1 μM, 37 °C, Molecular Probes, λ*_ex_*/λ*_em_* at 579/599 nm,. Before imaging, cells were washed twice with PBS. Lastly, the cells on coverslips were immediately imaged using a BZ-X800E fluorescence microscope (Leica Microsystems, Wetzlar, Germany) under DAPI and RHOD channels, respectively.

### 4.8. Statistical Analysis

In the histogram, the data represent mean ± standard deviation (SD) from at least three independent experiments. Differences at *p* < 0.05 were considered significant.

## 5. Conclusions

This study demonstrates that microwave-synthesized CNDs using the precursors ethylenediamine and citric acid can plausibly penetrate into the nucleus of cells (human A549 cells), and the CND penetration at different incubation times and concentrations was investigated. In contrast to earlier studies using fluorescence imaging techniques, the reported study was conducted with enhanced dark-field imaging technology and hyperspectral microscopy measurements to quantitatively determine the light signals in the nucleus reflected/scattered by the CNDs. Both experimental results and an analytical modeling analysis suggest that the CND penetration proceeds in a three-stage concentration-dependent manner with a half saturation incubation concentration of 479 μg/mL and achieves a penetration saturation above 750 μg/mL for the studied CNDs. These findings may help to facilitate the development of the CNDs as drug delivery carriers or live cell imaging agents, and for other biomedical applications.

## Data Availability

Not applicable.

## Supplementary Materials

The following supporting information can be downloaded at: https://www.mdpi.com/article/10.3390/molecules27082437/s1, Figure S1: A representative height profile from the AFM image; Figure S2: A representative TEM image with associated size profile analyses; Figure S3: Survey XPS spectrum analysis (A) and XPS spectra of N 1s (B) and O 1s (C); Figure S4: The photobleaching experiment under Xe lamp irradiation within 30 min; Figure S5: Light intensity measurements of cells without CNDs for different times, 6 h (A) and 24 h (B), and data analysis (C); Figure S6: The cell cytotoxicity measurement by the MTT assay in A549 cells incubated with different concentrations of CNDs. The error bars represent standard deviation. * Stands for a significant difference from the control (0 mg/mL) (p < 0.05); Figure S7: Confocal images (60×) of (left column) Mitotracker Red-stained cells, (middle column) fluorescence of the CNDs in cells at different concentrations (0, 0.4, 0.8 mg/mL incubation), and (right column) merged Mitotracker Red and fluorescence of CNDs in cells.

## Sample Availability

Samples of the compound CNDs are available from the authors.
