# Supplementary material for: Dark-Field Microscopic Study of Cellular Uptake of Carbon Nanodots: Nuclear Penetrability
Source: Molecules. 2022 Apr 9;27(8):2437. doi: 10.3390/molecules27082437 (PMC9032144; doi:10.3390/molecules27082437)
Supplement: Supplementary file 1 [file molecules-27-02437-s001.zip › molecules-1662426-supplementary.pdf]

Supplementary Materials

Dark-Field Microscopic Study of Cellular Uptake of Carbon Nanodots: Nuclear Penetrability

Wendi Zhang,[1] *Zuowei Ji,*[1] *Zheng Zeng,*[1] *Anitha Jayapalan,*[1] *Bhawna Bagra,*[1] *Alex Sheardy,*[1] *Peng He,*[2] *Dennis R LaJeunesse,*[1] *Jianjun We*[1*]

[1] Department of Nanoscience, Joint School of Nanoscience and Nanoengineering, University of North Carolina at Greensboro, Greensboro, NC 27401, United States.

[2] Department of Chemistry, North Carolina Agricultural and Technical State University, Greensboro, North Carolina 27411, United States

[*]To whom correspondence should be addressed.

Jianjun Wei, j_we@uncg.edu

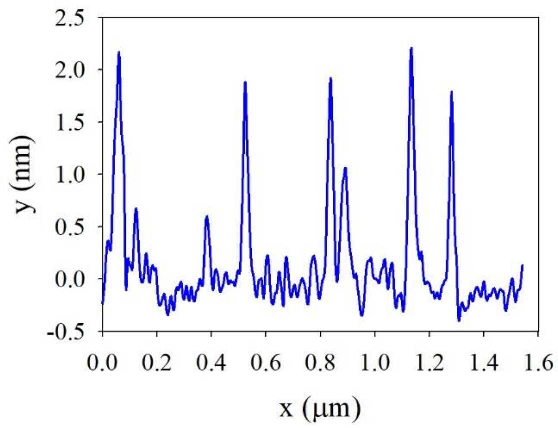

Figure S1. A representative height profile from the AFM image.

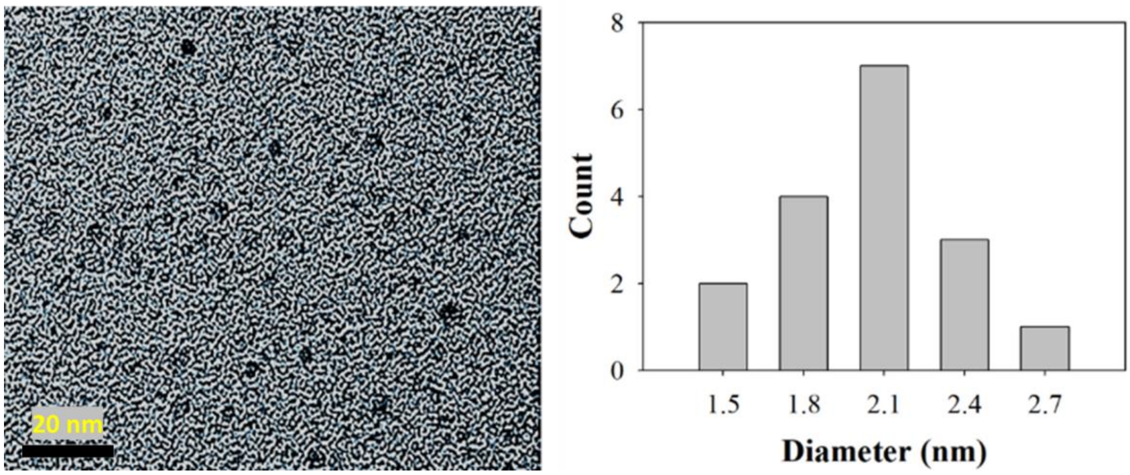

Figure S2. A representative TEM image with associated size profile analyses.

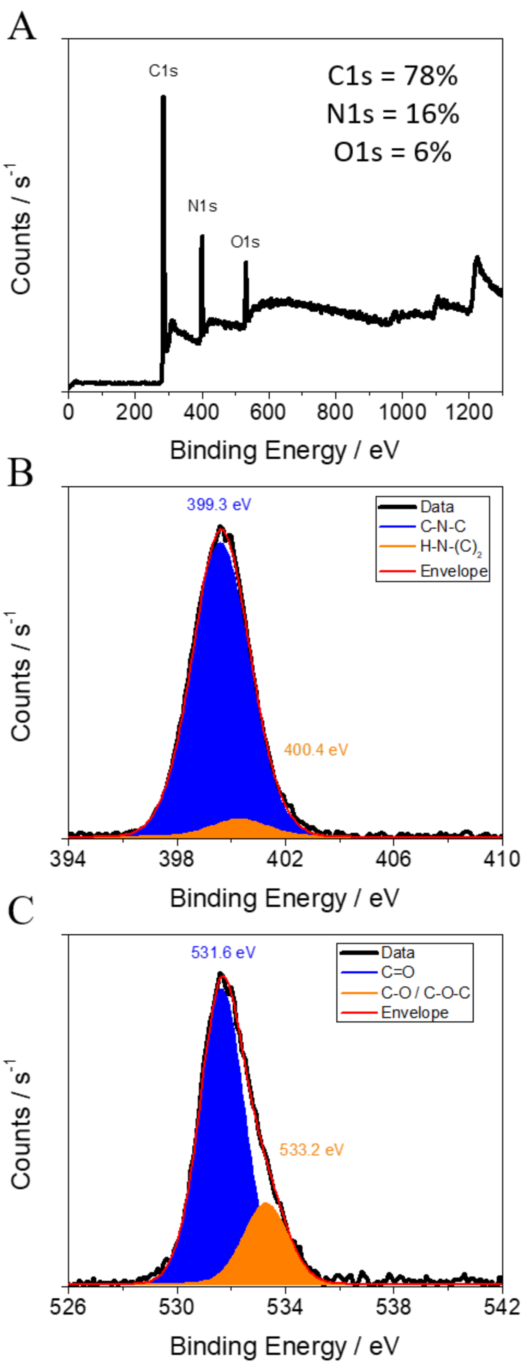

Figure S3. Survey XPS spectrum analysis (A) and XPS spectra of N 1s (B) and O 1s (C).

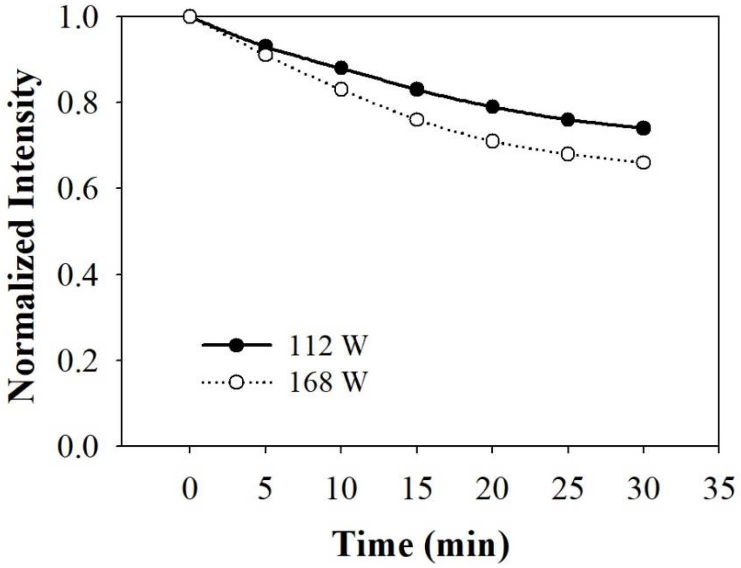

Figure S4. The photobleaching experiment under Xe lamp irradiation within 30 min.

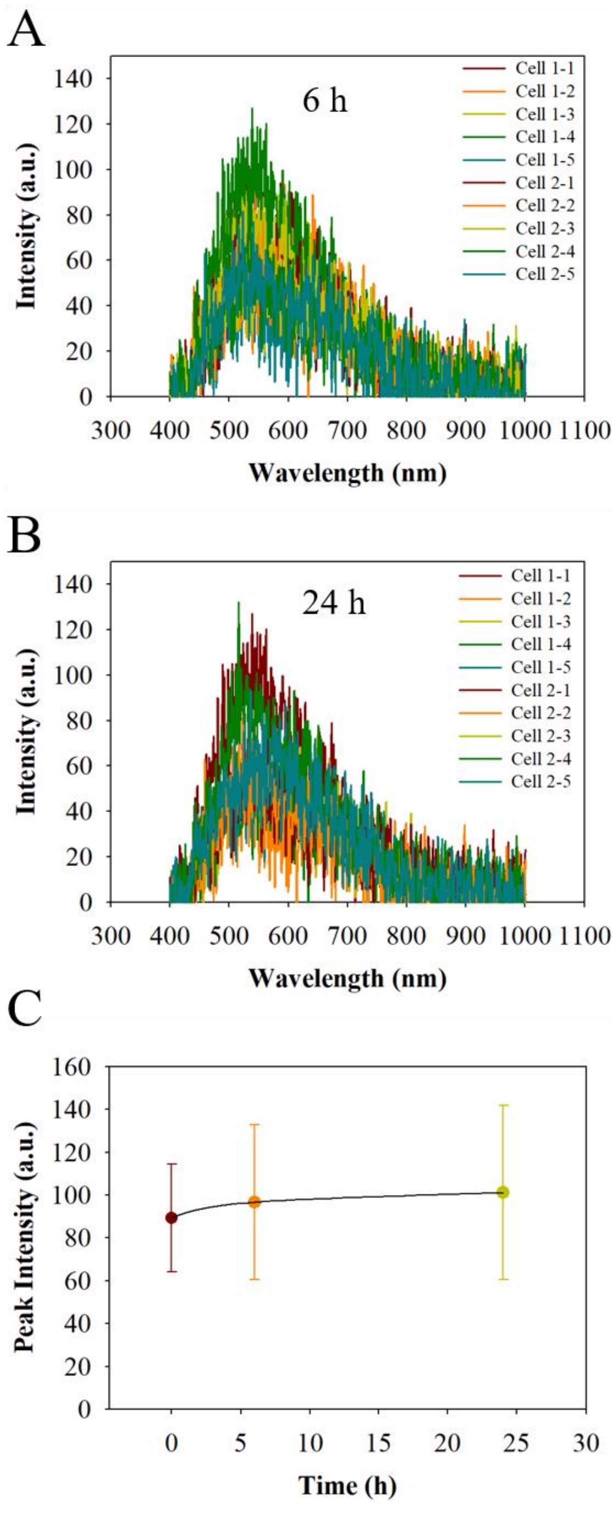

Figure S5. Light intensity measurements of cells without CNDs for different times: 6 h (A) and 24 h (B) and data analysis (C).

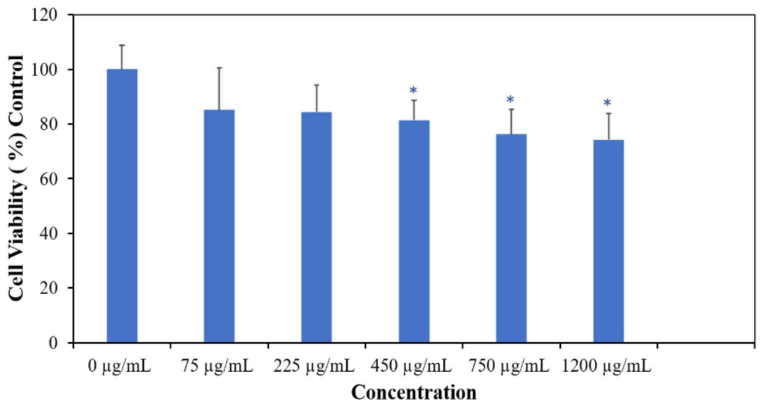

Figure S6. The cell cytotoxicity measurement by the MTT assay in A549 cells incubated with different concentrations of CNDs, The error bars represent standard deviation. *stands for a significant difference from the control (0 mg/mL) ($p < 0.05$).

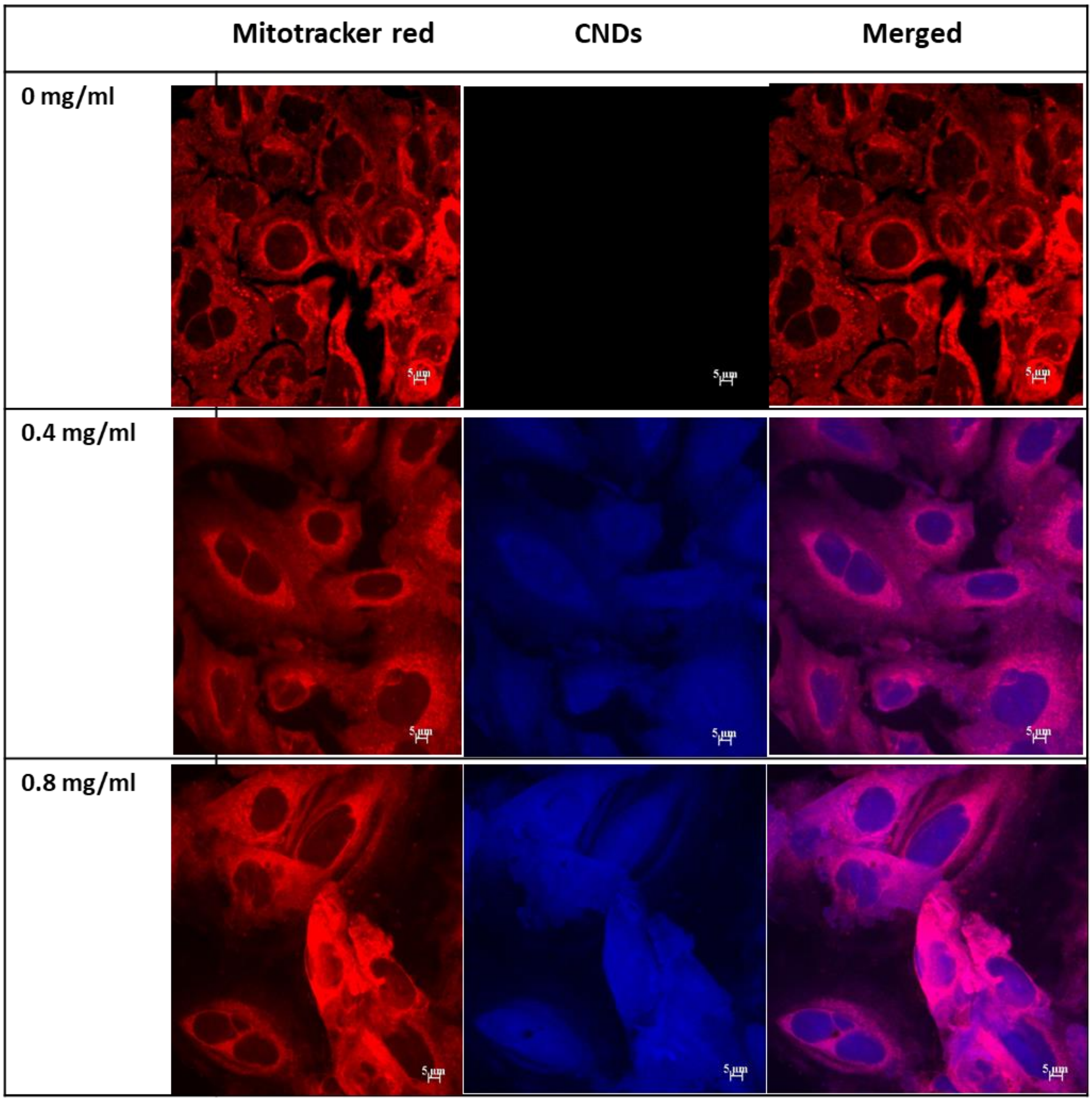

Figure S7. Confocal images (60x) of (left column) Mitotracker Red stained cells, (middle column) fluorescence of the CNDs in cells at different concentration (0, 0.4, 0.8 mg/mL incubation), and (right column) merged Mitotracker Red and fluorescence of CNDs in cells.

As the CNDs are blue fluorescent, DAPI dye is not chosen to stain the nuclear area. Mitotracker Red is used to label the mitochondria in order to discriminate the nuclear region inside cells. It is confirmed the intracellular distribution of the CNDs the nuclear region and some in the mitochondria of the A549 cells.
